# Post-translational modifications of na+/K+-atpase subunits: regulatory mechanisms and functional implications

**DOI:** 10.3389/fcell.2026.1913410

**Published:** 2026-09-03

**Authors:** Jia Tong, Liuyang Qin, Yunge Zhang, Weizhen Liu, Jie Liu, Chengbiao Lu

**Affiliations:** 1 The Second Affiliated Hospital of Xinxiang Medical University, Henan Key Laboratory of Biological Psychiatry (Henan Medical University), Xinxiang, Henan, China; 2 Institute of Psychiatry and Neuroscience, Henan Medical University, Xinxiang, Henan, China; 3 Henan Medical Key Laboratory for Research of Trauma and Orthopedics, The Third Affiliated Hospital of Henan Medical University, Xinxiang, Henan, China

**Keywords:** crosstalk, FXYD, Na+/K+-ATPase, post-translational modifications, protein function

## Abstract

**Background:**

The Na+/K+-ATPase (NKA), commonly referred to as the sodium pump, is a ubiquitous membrane protein that maintains cellular homeostasis by expelling three sodium ions from the cell and importing two potassium ions using ATP hydrolysis. This enzyme is critical for maintaining resting membrane potential, generating action potentials, and facilitating substance absorption and gland secretion. Additionally, NKA plays a significant role in overall homeostasis and has been implicated in tumor cell regulation.

**Aim of Review:**

This review aims to provide a comprehensive overview of the post-translational modifications (PTMs) that modulate the function, stability, and intracellular localization of NKA. These PTMs, including phosphorylation, glycosylation, palmitoylation, ubiquitination, and glutathionylation, have emerged as key regulators of NKA activity. By exploring these modifications, we seek to uncover novel insights into the dynamic regulation of NKA and its role in various physiological processes and disease states.

**Key Scientific Concept of Review:**

Recent research has unveiled the intricate interplay between different PTMs of NKA, highlighting their independent and synergistic effects on enzyme function. These modifications not only influence NKA’s ion transport capabilities but also interact with other regulatory pathways, thereby playing a crucial role in health and disease. This review will delve into the specific types of PTMs identified on NKA subunits, their functional consequences, and the potential for these modifications to serve as therapeutic targets. By synthesizing current knowledge and identifying gaps in understanding, this review aims to advance the field and inspire future research directions.

## 1 Introduction

NKA, first conceptualized by Jens Skou, is a member of the P-type ATPase superfamily (Palmgren, 2023). P-type ATPases are ATP-driven pumps that use ATP hydrolysis energy to transport ions cross the cytoplasmic membrane against a concentration gradient (Kühlbrandt, 2004). These ATPases participate in a multitude of biological processes, including the transport of sodium and potassium ions (e.g., NKA) (Ren et al., 2024; Clarke and Fan, 2011), calcium ions (e.g., SERCA pumps) (Zádor, 2023), protons (e.g., H+ -ATPase) (Falace et al., 2024), and heavy metal ions (Argüello et al., 2007; Ray and Gaudet, 2023), among others.

The NKA is a multi-subunit enzyme composed of catalytic α (approximately 113 kDa), glycoprotein β (ranging from 31 to 35 kDa) subunits, and a member of a FXYD family (Skou, 2004; Yap et al., 2021). The fundamental functional unit consists of α and β subunits forming a macromolecular complex, with four isoforms of the α subunit (α1-4), three of the β subunit (β1-3) available (Sweadner, 1989; Dhalla et al., 2024), and also a novel β (β4) in all vertebrate (Pestov et al., 2007). The α-subunit, serving as the catalytic core of the enzyme, is responsible for the transport of cations and contains the binding sites for Na+, K+, ATP, and cardiotonic steroids such as ouabain (Howie et al., 2013). It requires obligatory association with a β-subunit to traffic through the secretory pathway to the plasma membrane. The FXYD family in mammals comprises seven distinct members, including FXYD1, known as phospholemman (Cheung et al., 2013); FXYD2, referred to as the γ subunit; FXYD3, identified as the mammary tumor marker Mat-8; FXYD4, termed the corticosteroid hormone-induced factor; FXYD5, associated with the ion channel RIC or dysadherin; FXYD6, recognized as phosphohippolin; and FXYD7 (Béguin et al., 2002; Geering, 2006). FXYD proteins serve as auxiliary subunits, associating with the α-subunit of the NKA and influencing the apparent affinity of the Na+/K+ and affect the maximal turnover rate (Vmax) of NKA (Yap et al., 2021), thereby playing a crucial role in maintaining cellular ion homeostasis and adapting to physiological and pathological changes (Geering, 2005).

PTMs are essential mechanisms used by cells to regulate protein function and are highly dynamic, allowing for rapid adaptation to environmental changes. PTMs can modulate various aspects of protein function, including stability, localization, and interaction with other molecules (Lee et al., 2023). A substantial body of research indicates that the activity and function of NKA can be regulated by PTMs, such as phosphorylation, glycosylation, palmitoylation, and others. PTMs of NKA are crucial for its function and regulation and are associated with a variety of diseases. Abnormal phosphorylation of specific NKA subunits in certain neurodegenerative diseases may be related to the disruption of intracellular signaling pathways. Additionally, the development of cardiovascular diseases, kidney diseases, and some cancers is linked to the state of the NKA’s PTMs. This review will describe the types of PTMs that the subunits of NKA undergo and their functional implications.

## 2 Phosphorylation

Phosphorylation is a common PTM that involves the addition of a phosphate group to serine, threonine, or tyrosine residues on a protein (Khoury et al., 2011). Various subunits of NKA possess amino acid sites that are subject to phosphorylation, and the phosphorylation of these subunits plays a key role in regulating the activity of NKA.

**FIGURE 1:**
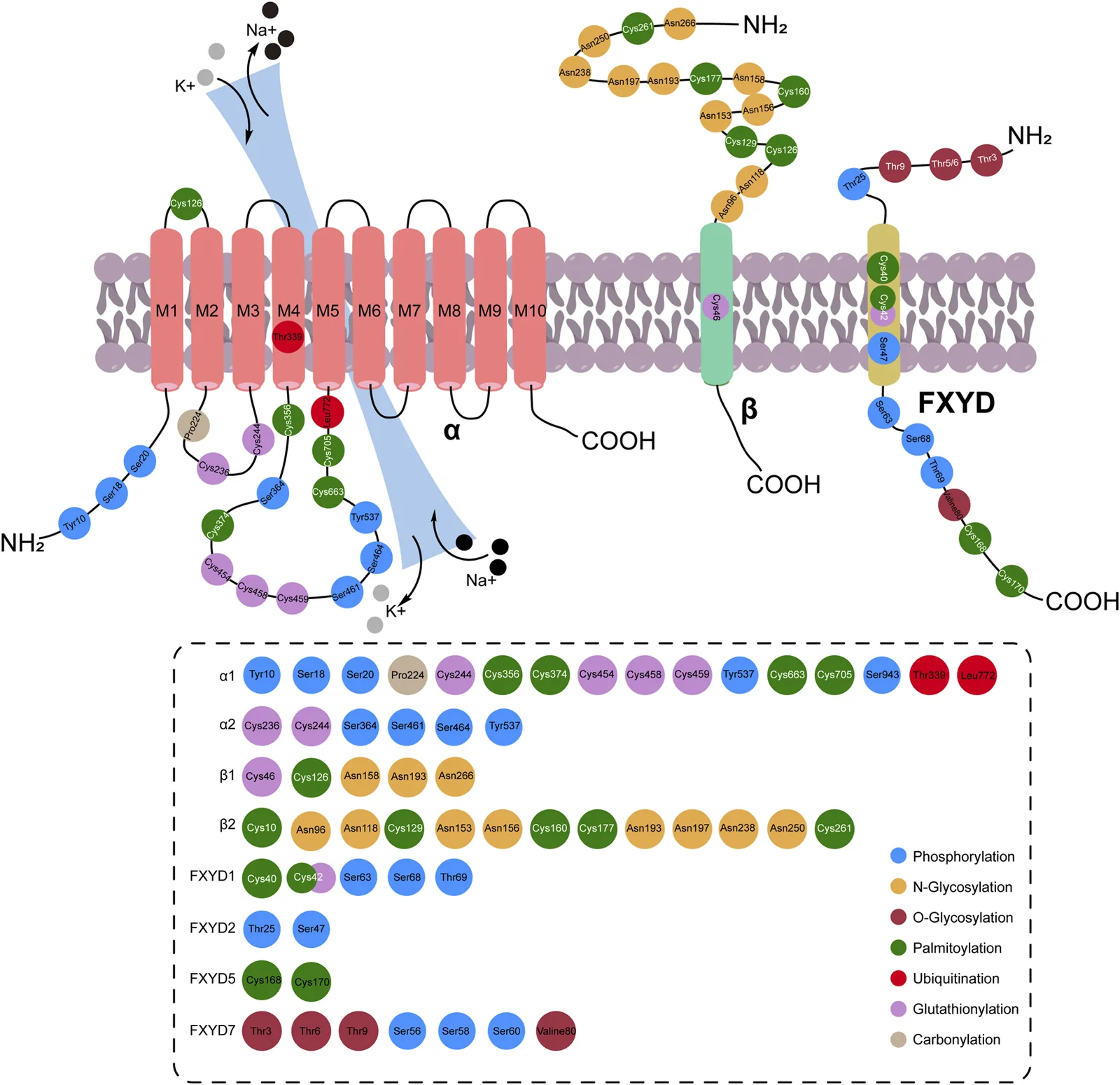
Summary of reported PTMs on specific sites of diverse NKA subtypes. M1-M10: transmembrane domains 1–10.

The α subunit can undergo phosphorylation on serine/threonine residues via protein kinase A/C (PKA/PKC) or on tyrosine residues via a tyrosine kinase-dependent mechanism. When it comes to specific isoforms (as summarized in Table 1), PKA directly phosphorylates the α1 subunit at Ser943 (Fisone et al., 1994), while other studies have shown that PKC-ζ also phosphorylates the α1 subunit at Ser18 and Ser20 (Dada et al., 2003; Chibalin et al., 1999) (Figure 1). Phosphorylation of the α1 subunit by PKA consistently results in the inhibition of NKA activity. Similar to PKA-mediated phosphorylation, phosphorylation by PKC of α1 subunit also leads to inhibition of NKA activity (Vasilets et al., 1990; Middleton et al., 1993). The α2 subunit can be phosphorylated at Ser364 (Coppi and Guidotti, 1997a). A mutation at this position not only reduces the NKA activity but also leads to NKA retention in the ER (Coppi and Guidotti, 1997a). Sodium cyanide exposure induces the phosphorylation of α2 at Ser464 by PKC-ε, which leads to a decrease in NKA activity (Cheung et al., 2019). Liu JG et al. have reported that short-term morphine treatment leads to a significant reduction in the total phosphorylation levels of the α3 subunit of NKA in the striatum (Wu et al., 2007). However, *in vivo* long-term morphine treatment has been shown to decrease striatal NKA activity through the enhancement of PKA activity (Wu et al., 2007).

**TABLE 1:** PTMs of NKA: subunits, enzymes, sites, and functional impact.

PTMs	Subunits	Enzyme	Modification sites	Functions on NKA
Phosphorylation	α1	PKA; PKCζ; Src	Ser18/20 (Dada et al., 2003; Chibalin et al., 1999)/943 (Fisone et al., 1994) Tyr10 (Féraille et al., 1999)/537 (Doné et al., 2002)	Inhibition (Fisone et al., 1994) or downregulation (Vasilets et al., 1990; Middleton et al., 1993; Chibalin et al., 1997), (Doné et al., 2002) of the NKA activity
	α2	PKCε; Src	Ser364 (Coppi and Guidotti, 1997a)/461/464 (Cheung et al., 2019) Tyr537 (Al-Khalili et al., 2003)	Inhibition of NKA activity by PKC (Cheung et al., 2019), causes NKA retention in the ER by Src (Al-Khalili et al., 2003)
	α3	PKA; Lyn, Yes, Agrin	Unknown	Inhibition of NKA activity by PKA (Wu et al., 2007); activating the pump activity by Lyn or Yes (Wang and Yu, 2005),while inhibiting the pump activity by Agrin (Hilgenberg et al., 2009; Hilgenberg et al., 2006; Hilgenberg and Smith, 2004)
	α4, β1-3	Unknown	Unknown	Unknown
	FXYD1	PKA	Ser68 (Despa et al., 2005;Silverman et al., 2005)	Increasing Na affinity, increasing Vmax, activating the NKA (Silverman et al., 2005)
		PKC	Ser63/68 (Bossuyt et al., 2009), Thr69 (Fuller et al., 2009)	Increasing Vmax with no change in Na affinity (Han et al., 2006; Fuller et al., 2009)or with increased in Na affinity (Bossuyt et al., 2009)
		NIMA kinase	Ser63 (Lu et al., 1994)	Activation of NKA via phosphorylating FXYD1 (Lu et al., 1994)
		DMPK	Unknown	Unknown
	FXYD2	PKA, PKC	Ser47 (Cortes et al., 2006), Thr25 (Cortes et al., 2011)	Activation of NKA (Cortes et al., 2011; Cortes et al., 2006)
	FXYD3-6	Unknown	Unknown	Unknown
	FXYD7	Unknown	Ser56/58/60 (Crambert et al., 2004)	No effect on NKA (Crambert et al., 2004)
Dephosphorylation	α1	PP2A	Ser18 (Lecuona et al., 2006)	Recruitment to the cell plasma membrane (Lecuona et al., 2006)
		PP1	Unknown	Activation of NKA (Ragolia et al., 1997)
		Calcineurin	Unknown	Activation of NKA (Ibarra et al., 2002; Mallick et al., 2000)
	α2-4, β1-3	Unknown	Unknown	Unknown
	FXYD1	PP1, PP2A	Ser63/68 (El-Armouche et al., 2011); Thr69 (El-Armouche et al., 2011)	Inhibition of the NKA via dephosphorylating FXYD1 (El-Armouche et al., 2011)
	FXYD2-7	Unknown	Unknown	Unknown
N-glycosylation	α1-4	Unknown	Unknown	Unknown
	β1	Unknown	Asn158/193/266 (Vagin et al., 2006)	No significant effect on NKA (Vagin et al., 2006); regulating cell–cell adhesion (Vagin et al., 2006; Vagin et al., 2007)
	β2	Unknown	Asn96/118/153/156/193/197/238/250 (Tokhtaeva et al., 2010; Roldán et al., 2022)	Maturation and membrane targeting of NKA, affecting the assembly of the α1-β2 complex (Tokhtaeva et al., 2010); regulating cell–cell adhesion (Roldán et al., 2022)
​	β3	Unknown	Unknown	Unknown
	FXYD1-7	Unknown	Unknown	Unknown
O-glycosylation	FXYD5	O‐glycosidase (Hotta et al., 2023)	Unknown	Disrupts the cell-cell trans dimerization of NKA (Tokhtaeva et al., 2016)
	FXYD7	Neuraminidase, O‐glycosidase (Béguin et al., 2002)	Valine80 (Crambert et al., 2004); Thr3/6/9 (Béguin et al., 2002)	No impact on the NKA (Béguin et al., 2002)
Palmitoylation	α1-4, β1-3	Unknown	Unknown	Unknown
	FXYD1	zDHHC5	Cys40/42 (Tulloch et al., 2011)	Inhibition of NKA (Howie et al., 2013; Tulloch et al., 2011; Main et al., 2022)
	FXYD5	Unknown	Cys168/170 (Martin et al., 2011)	Unknown
	FXYD2-4,7	Unknown	Unknown	Unknown
Depalmitoylation	α1	PPT1	Cys374/705 (Yang et al., 2010); Cys356 and Cys663 (Forrester et al., 2011)	Perturbs pump’s activity (Yang et al., 2010)
	α2	PPT1	Unknown	Unknown
	α3	PPT1	Unknown	Unknown
	α4	Unknown	Unknown	Unknown
	β1	PPT1	Cys126 (Gorenberg et al., 2022)	Unknown
	β2	PPT1	Cys10/129/160/177/261 (Gorenberg et al., 2022; Collins et al., 2017)	Stabilization of β-sheets that form the channel pore (Gorenberg et al., 2022); regulation of neuron–astrocyte adhesion via depalmitoylation (Gorenberg et al., 2022)
	β3	Unknown	Unknown	Unknown
	FXYD5	Unknown	Cys168/170 (Lubarski Gotliv, 2016)	Unknown
	FXYD1-4,6,7	Unknown	Unknown	Unknown
Ubiquitination	α1	ZNRF1, ZNRF2	Thr339, Leu772 (Hoxhaj et al., 2012)	Regulation of cell surface expression of NKA (Hoxhaj et al., 2012)
	α2-4	Unknown	Unknown	Unknown
	β1	RNF183	Unknown	Degradation (Okamoto et al., 2020)
	β2-3	Unknown	Unknown	Unknown
	FXYD1-7	Unknown	Unknown	Unknown
Glutathionylation	α1	Glutaredoxin, dithiothreitol Oxidized glutathione	Cys244/454/458/459 (Petrushanko et al., 2012)	Glutathionylation decreases Na + pump’s activity, while deglutathionylation restores the activity (Petrushanko et al., 2012; Fuller et al., 2003)
	α2	Oxidized glutathione	Cys236/244 (Petrushanko et al., 2012)	
	α3,4	Unknown	Unknown	Unknown
	β1	Glutaredoxin 1, peroxynitrite, hydrogen peroxide	Cys46 (Liu et al., 2012)	Destabilize the interaction between cardiac α- and β-subunits (Figtree et al., 2009); modest decrease the Vmax of the pump (Figtree et al., 2009)
	β2,3	Unknown	Unknown	Unknown
	FXYD1	Unknown	Cys42 (Bibert et al., 2011)	Increase Vmax (Bibert et al., 2011)
	FXYD2-7	Unknown	Unknown	Unknown

On the other hand, PKA and PKC phosphorylations do not fully account for the basal phosphorylation level of the α subunit observed in various cells (Beguin et al., 1994; Carranza et al., 1996). Other reports recognize the α1 subunit as a substrate for the nonreceptor tyrosine kinase c-Src, which can be phosphorylated at Tyr10 in response to insulin stimulation, thereby controlling the sodium reabsorption in the proximal convoluted tubule (Féraille et al., 1999). While, phosphorylation at Tyr537 of α1 is essential for endocytosis (Ogimoto et al., 2000; Doné et al., 2002). Tyr537 is also predicted as the tyrosine phosphorylation target of α2 (Al-Khalili et al., 2003), though the specific mechanism involved in this modification, as well as the broader implications for the regulation of the Na+ pump, require further investigation. The a3 subunit can also be directly phosphorylated by tyrosine kinases. Particularly, Lyn, a member of the Src family of tyrosine kinases, have been identified to bind directly to the a3 subunit of NKA, thereby activating the pump current (Wang and Yu, 2005). Agrin, served as a tyrosine kinase, specifically bind to the α3 subunit, leading to its tyrosine phosphorylation, which in turn inhibits the activity of the NKA (Hilgenberg et al., 2009; Hilgenberg et al., 2006; Hilgenberg and Smith, 2004).

FXYD1, initially discovered as a predominant phosphoprotein within the cardiac sarcolemma, was soon acknowledged as the key substrate for both PKA and PKC in cardiac tissue (Presti et al., 1985a; Presti et al., 1985b). Belonging to the FXYD family of regulatory proteins that govern pump activity (Sweadner and Rael, 2000), FXYD1 is found in complex with the cardiac Na+ pump, where it alters the pump’s transport properties (Bossuyt et al., 2005; Bossuyt et al., 2009; Crambert et al., 2002; Fuller et al., 2004; Bossuyt et al., 2006). Unphosphorylated FXYD1 inhibits the cardiac Na+ pump by reducing its Na+ affinity (Bossuyt et al., 2009; Despa et al., 2005; Han et al., 2009; Han et al., 2006) and Vmax (Fuller et al., 2004; Fuller et al., 2009; Silverman et al., 2005; Pavlović et al., 2007), and phosphorylation of FXYD1 by PKA (on Ser68) or PKC (on Ser63/68 and Thr69) relieves this inhibition (Bossuyt et al., 2009; Despa et al., 2005; Fuller et al., 2009) and in some models actually increases Vmax of the Na+ pump (Fuller et al., 2004; Han et al., 2009; Pavlović et al., 2007). Phosphorylating FXYD1 to activate the cardiac Na+ pump is essential for safeguarding the heart against arrhythmias triggered by excessive catecholamine-induced Na+ and Ca2+ overload *in vivo* (Despa et al., 2008). Additionally, the phosphorylation of FXYD1 affects the function of the α subunit. Studies have indicated that both PKA and PKC phosphorylation of FXYD1 increase the Na+ affinity for the α1 and α2 isoforms (Bossuyt et al., 2009), with PKC-specifically elevating the Vmax for α2-containing pumps (Bibert et al., 2008).

FXYD1 is also an excellent substrate for Never-In-Mitosis-A (NIMA) kinase, which phosphorylates Ser63 (Lu et al., 1994). The NIMA kinase is a member of the serine/threonine kinase family, characterized as a cell cycle-regulated protein kinase (Lu et al., 1994; Lu et al., 1993). FXYD1 phosphorylation mediated by NIMA kinase activates the NKA (Lu et al., 1994). Additional research has confirmed that FXYD1 is a suitable substrate for myotonic dystrophy protein kinase (DMPK) *in vitro* (Mounsey et al., 2000). However, similar to the case with NIMA kinase, the specific functional implications of DMPK-mediated FXYD1 phosphorylation on KNA are not yet fully understood and necessitate further exploration. This underscores the need for a comprehensive study of FXYD1’s phosphorylation by different kinases and its subsequent effects on cellular functions.

FXYD2 is a small polypeptide of 58 amino acids with the molecular weight of 6.5–10 kDa, which co-purifies and co-localizes with the α- and β-subunits of the pig kidney outer medulla NKA (Therien et al., 1997; Therien and Blostein, 2000; Fontes et al., 1999; Füzesi et al., 2005). Some recent findings have implicated the FXYD2 as a modulator of the NKA, through different effects: by an increase of Na+ apparent affinity when K+ concentration is high, resulting in an increase of K+/Na+ competition at cytoplasmic Na+ sites; and by an increase of the apparent affinity for ATP (Pu et al., 2001; Zouzoulas et al., 2003). Focusing on the phosphorylation, FXYD2 can be phosphorylated by membrane-anchored PKA (Kurihara et al., 2000) at Ser47 and PKC at Thr25 (Cortes et al., 2011), and the phosphorylation of FXYD2 can activate the function of NKA.

For FXYD3-6, although there are currently no reports on their specific mechanisms of phosphorylation, they exert multifaceted functions in regulating NKA’s activity. The extracellular and transmembrane domains of FXYD3 are homologous to those of FXYD1. Unlike FXYD1, the cytoplasmic domain of FXYD3 lacks the consensus phosphorylation sites typically found in FXYD1. Interestingly, when FXYD3 is expressed in *Xenopus oocytes*, it induces hyperpolarization-activated chloride currents, a phenomenon also observed with FXYD1 expression (Moorman et al., 1992). This suggests that despite the differences in their cytoplasmic domains, FXYD3 and FXYD1 may share some functional similarities in modulating ion channel activities. Both FXYD4 and FXYD2 can associate with the NKA and modulate its transport properties (Béguin et al., 2001). The FXYD4 increases while FXYD2 decreases the apparent Na+ affinity of the NKA (Béguin et al., 2001). The FXYD5 has been found to promote chronic inflammatory responses (Song et al., 2022), and is associated with tumor progression, including breast cancer and renal cell carcinoma (Nam et al., 2006; Schüler et al., 2012). FXYD6 and FXYD7 seem to be exclusive to the nervous system (Béguin et al., 2002; Delprat et al., 2007; Kadowaki et al., 2004). FXYD6 can modulate human NKA function by accelerating both the Na+-deocclusion step and the pump turnover rates (Meyer et al., 2020).

The activity and subcellular distribution of NKA are governed not only by kinases but also by opposing dephosphorylation events mediated by serine/threonine protein phosphatases. Three phosphatases have been implicated in NKA dephosphorylation: PP1, PP2A, and calcineurin.

PP1 mediates insulin-induced dephosphorylation of the α1 subunit in skeletal muscle, leading to pump activation. This effect is abolished by PP1 inhibitors but not by PP2A-selective inhibitors, and is enhanced by overexpression of the PP1 regulatory subunit PP1G, confirming PP1 as the primary phosphatase in this context (Ragolia et al., 1997). PP2A directly dephosphorylates the α1 subunit at Ser18 in alveolar epithelial cells following dopamine receptor stimulation. This modification is essential for NKA recruitment from intracellular compartments to the plasma membrane, as blocking PP2A prevents both dephosphorylation and membrane insertion (Lecuona et al., 2006). Calcineurin, a calcium/calmodulin-dependent phosphatase, dephosphorylates α1 subunit in renal proximal tubules in response to α-adrenergic stimulation, thereby increasing pump activity. Inhibition of calcineurin enhances NKA phosphorylation (Ibarra et al., 2002). In the brain, calcineurin mediates norepinephrine-induced NKA activation through α_1A_-adrenoceptor coupled calcium signaling (Mallick et al., 2000).

Interesting, PP1 and PP2A are involved in the dephosphorylation of FXYD1, and it therefore provides a functional link between both these phosphatases and the NKA (Neumann et al., 1999). Further findings indicate that Ser68 (and potentially Thr69) positions of FXYD1 are substrates for PP1, and Ser63 is likely targeted by PP2A (El-Armouche et al., 2011). FXYD1 dephosphorylation tends to inhibit the activity of NKA (El-Armouche et al., 2011).

Collectively, these studies demonstrate that NKA dephosphorylation is not a passive reversal of kinase activity but an actively regulated process mediated by distinct phosphatases, such as PP1, PP2A, and calcineurin. Through dephosphorylation of specific residues, these enzymes regulate NKA trafficking, membrane abundance, and catalytic activity, with direct consequences for ion transport and systemic sodium homeostasis.

## 3 Glycosylation

Glycosylation primarily occurs in two forms: N-linked and O-linked glycosylation. N-glycosylation is the process of attaching sugar molecules to the asparagine (Asn) residues of proteins. The NKA is capable of undergoing both N-linked and O-linked glycosylation modifications. Glycosylated NKA is transported to the Golgi apparatus, further processed, and then sent to the cell membrane, where it performs its ion pumping function. Moreover, changes in glycosylation may be related to certain disease states, such as cardiovascular diseases and tumors.

Research indicates that all β subunits of NKA are glycoproteins and possess 3–7 N-linked sugar chains (Geering, 1990), which serves as an important molecular chaperone to aid in the correct membrane insertion, stability, and trafficking of the α subunit to the plasma membrane (Geering, 2001). The extracellular domain has three N-linked glycosylation sites at Asn-158, Asn-193, and Asn-266 (Tokhtaeva et al., 2010; Pedemonte et al., 1990; Beggah et al., 1997; Miller and Farley, 1988) and three disulfide bonds that are conserved among all of the β isoforms (Laughery et al., 2003). Removal of all N-linked glycosylation of the β1 subunit, by replacing the three Asn residues at the points of sugar attachment with glutamines, produces no significant effect on NKA assembly or plasma membrane delivery (Laughery et al., 2003). However, the glycosylation of the β1 subunit serves to protect it from proteolytic degradation, thereby contributing to the stability of NKA to a certain extent (Laughery et al., 2003).

Inhibition of N-glycosylation in the β1 and β3 subunits by tunicamycin or mutation at the N-glycosylation sites has minimal impact on the assembly of the α1–β1 complex (Beggah et al., 1997; Zamofing et al., 1989), the pump’s transportation to the plasma membrane (Laughery et al., 2003; Vagin et al., 2006), and the activity of the NKA (Beggah et al., 1997; Laughery et al., 2003; Takeda et al., 1988). While the situation is markedly different for the β2 subunit. β2 subunit has eight glycosylation sites at Asn-96, Asn-118, Asn-153, Asn-156, Asn-193, Asn-197, Asn-238, and Asn-250 (Tokhtaeva et al., 2010). Mutation of these Asn sites or the use of inhibitors, such as treatment with tunicamycin, to obstruct glycosylation, can lead to the retention of the β2 subunit in the endoplasmic reticulum (ER) and hinder its trafficking to the Golgi apparatus, affecting the endogenous assembly of the β2 subunit with the α1 subunit of NKA (Tokhtaeva et al., 2010). This indicates that, unlike β1 and β3, the N-glycosylation of the β2 subunit plays a pivotal role in NKA’s trafficking and maturation processes (Tokhtaeva et al., 2010).

Besides, the β subunit glycosylation is often closely related to cell-to-cell adhesion functions. Both β1 and β2 subunits of NKA acts as the adhesive protein (Vagin et al., 2006; Gloor et al., 1990; Roldán et al., 2022). Substituting the three Asn residues of β1 with Gln significantly disrupts cell-to-cell adhesion, indicating that N-glycans attached to the β1 subunit are essential for maintaining cellular cohesion (Vagin et al., 2006). The β2 subunit of NKA was originally identified as the adhesion molecule on glia (AMOG) that mediates the adhesion of astrocytes to neurons to promote neurite outgrowth in the CNS (Roldán et al., 2022). Previous study has showed that glycosylated extracellular domain of β2/AMOG can make an energetically stable trans-interacting dimer, suggesting that N-glycosylation of β2 subunit is important for β2–β2 trans-interaction (Roldán et al., 2022), which is essential for establishing the cell-to-cell interaction.

However, the glycosylation process of the α subunit is considered to be unusual. As we know, O-linked glycoproteins of the ER (Abeijon and Hirschberg, 1988) and Golgi apparatus (Ca et al., 1988), as well as other membrane-associated proteins (Hart et al., 1989), typically arrange their carbohydrates to face the cytosolic side rather than the luminal side. However, the α subunit of the NKA is an exception, as this glycoprotein has its N-linked oligosaccharide moieties located on the cytosolic side of the cell membrane (Pedemonte et al., 1990). This observation challenges our current understanding of the synthesis mechanism for N-linked oligosaccharides (Shull et al., 1985). Furthermore, the specific mechanisms of α subunit N-glycosylation modifications and their regulatory effects on NKA have not yet been reported.

Certain FXYD proteins are capable of undergoing O-glycosylation modifications. As FXYD5 lacks sites for N-glycosylation, it can undergo O-glycosylation instead (Lubarski et al., 2007; Ino et al., 2002), a modification that is particularly relevant in the tumor context (Shimamura et al., 2003; Shimamura et al., 2004; Tsuiji et al., 2003). The extracellular serines, threonines, and prolines residues of FXYD5 are likely that some of these residues are O-glycosylated (Lubarski et al., 2007). FXYD7 is also identified as a potential target for O-glycosylation which appears to be important for protein stability (Béguin et al., 2002), with the specific sites of glycosylation occurring at threonine residues 3, five (or 6) and 9, and neuraminidase and O‐glycosidase can make effect on the electrophoretic mobility of FXYD7 (Béguin et al., 2002; Crambert et al., 2004). The C-terminal valine residue of FXYD7 may be critically involved in the rapid exit from the ER. Deletion of a C-terminal valine residue (Val80) in FXYD7 significantly delayed its O-glycosylation processing, retention and degradation in the ER, and retarded the rate of its cell surface expression (Crambert et al., 2004). While FXYD7 glycosylation is not essential for its impact on the K+ affinity of the NKA, nor does it affect the Na+ affinity of NKA (Béguin et al., 2002).

## 4 Palmitoylation

Palmitoylation is a reversible PTM where a palmitoyl group (C16:0) is covalently linked to the thiol group of cysteine residues at the C-terminus of a protein through a thioester bond (Aicart-Ramos et al., 2011). This modification plays a crucial role in governing protein stability and function, and is essential for the trafficking of proteins to the membrane (Greaves and Chamberlain, 2007) or specific membrane domains (Levental et al., 2010). Defects in the S-palmitoylation process of certain proteins have also been associated with a range of disorders (Milnerwood et al., 2013). *In vivo*, palmitoylation is thought to be catalyzed by a group of multi-spanning membrane proteins known as protein acyl transferases (Mitchell et al., 2006). These enzymes possess zinc-finger motifs and a characteristic aspartate–histidine–histidine–cysteine (zDHHC) domain architecture, which are instrumental in catalyzing the process (Lemonidis et al., 2015; Fukata et al., 2004). As protein palmitoylation is a reversible process, protein thioesterases, enzymes such as acyl-protein thioesterases (Duncan and Gilman, 1998; Kathayat et al., 2018), α/β hydrolase domain-containing 17 proteins (Yokoi et al., 2016; Tortosa et al., 2017), and palmitoyl-protein thioesterases (PPTs) (Koster and Yoshii, 2019) are responsible for the depalmitoylation process. They remove the palmitate group from proteins, thus completing the cycle of this dynamic and reversible PTM.

Proteomic studies have consistently reported on the palmitoylation of the NKA α1 subunit at specific cysteine residues (Dowal et al., 2011; Gorenberg et al., 2022). The pioneering work in this area pinpointed Cys374 with high confidence and Cys705 with moderate confidence on the α1-subunit, in addition to less certain sites at Cys211 and Cys249 (Yang et al., 2010). Both the high- and medium-confidence sites are located within the α1-subunit’s P-domain, close to the catalytic Asp376 that is phosphorylated during the pump’s operational cycle (Morth et al., 2007). The high-confidence site is particularly noteworthy for its proximity to this key residue. Conservation of these cysteines is evident across different species and heart-expressed isoforms. Although the palmitoylation at Cys374 could impact the α1 subunit’s structure and, by extension, the pump’s activity, the functional implications of this modification are still under investigation. Furthermore, other P-domain cysteines, Cys356 and Cys663, identified in another study as palmitoylated (Forrester et al., 2011), currently cannot have their regulatory effects on NKA determined due to limited literature.

All β subunits of the pump, with the exception of the β3 subunit, are considered to be palmitoylated, as the β3 subunit lacks the cysteine residues necessary for palmitoylation (Martin et al., 2011; Kang et al., 2008). The β1 and β2 subunits each contain three highly conserved disulfide bonds within their extracellular domains, which are critical for the structural and functional maturation of the pump (Zakipour and Alemzadeh, 2021). The specific palmitoyltransferase responsible for the palmitoylation of these subunits has not been clearly identified.

In terms of depalmitoylation, based on evidence from recent proteomic studies, α1-3 and β1-2 subunits were identified as high-confidence PPT1 substrates (Gorenberg et al., 2022; Senner et al., 2003). Depalmitoylation of the β2 subunit, mediated by PPT1, enhances the stability of the β2 subunit heterodimer through the regulation of disulfide bonds. Similar to β2, the β1 and α subunits also undergo persistent palmitoylation at Cys126 in the context of PPT1 deficit status (Gorenberg et al., 2022). However, the role of this modification in regulating NKA function remains unknown (Gorenberg et al., 2022), and it is also worth further investigation to identify other PPTs that catalyze the depalmitoylation of these subunits, beyond PPT1.

Given that all FXYD members possess at least one conserved cysteine residue, it is plausible that palmitoylation may represent a general regulatory mechanism for this family, although experimental evidence currently supports this modification only for FXYD1 and FXYD5. FXYD1 is recognized to be palmitoylated at Cys40 and Cys42 (Tulloch et al., 2011) by zDHHC5 (Plain et al., 2020; Main et al., 2022), an palmitoyl acyltransferase enzyme characterized by a cysteine-rich region with a conserved Asp-His-His-Cys motif (Mitchell et al., 2006) within the active site (Jennings and Linder, 2012).

Functional studies on FXYD1 palmitoylation have yielded partially conflicting observations. Unpalmitoylated FXYD1 degrades more rapidly than the wild-type protein in transiently transfected cells, whereas palmitoylation of FXYD1 has been reported to inhibit the Na+ pump (Tulloch et al., 2011; Main et al., 2022). Treatment with the palmitoyl acyltransferase inhibitor 2-bromopalmitate reverses this inhibitory effect, suggesting that palmitoylation may shift FXYD1 from an activator toward an inhibitor of the Na+ pump (Howie et al., 2013). However, the functional consequence of FXYD1 palmitoylation may be more nuanced than a simple inhibitory switch. Computational modeling has proposed that palmitate incorporation at Cys42 may exert an inhibitory effect by orienting helix 3 of FXYD1 over the sodium-binding site on the α subunit, whereas palmitoylation at Cys40 could potentially facilitate pump activity by exerting force in the opposite direction (Fuller et al., 2013) (Hershko and Ciechanover, 1998). This raises the possibility that individual palmitoylation sites within the same protein may exert opposing effects, although these predictions await experimental validation.

FXYD5 has also been validated to undergo palmitoylation at Cys168 and Cys170 (Martin et al., 2011). Neither residue is exclusive for FXYD5 and both are located in equivalent positions throughout the FXYD family. Although the equivalent cysteines in all FXYD proteins are predicted to undergo palmitoylation, this modification has been demonstrated experimentally only for FXYD1 and FXYD5 (Martin et al., 2011; Tulloch et al., 2011). The functional role of FXYD5 palmitoylation, if any, has not yet been characterized. Nevertheless, since all FXYD members have at least one conserved cysteine residue, palmitoylation may be a general means of regulating the pump.

In summary, current evidence suggests that palmitoylation of FXYD1 is associated with inhibition of NKA activity, yet the limited number of studies, the potential for site-specific opposing effects, and the complete lack of functional data for other FXYD members preclude a definitive conclusion regarding the net directionality of this modification. The physiological significance of palmitoylation across different NKA isoforms and tissue contexts, as well as its potential role as a general regulatory mechanism for the FXYD family, remain to be established through further investigation.

## 5 Ubiquitination

Ubiquitination is a PTM that marks proteins for various cellular functions, including degradation. This process is regulated by a cascade of enzymes—E1 activating, E2 conjugating, and E3 ligating enzymes—which work together to attach ubiquitin in a highly specific manner to target proteins (Hershko and Ciechanover, 1998; Schulman, 2011). Coppi and Guidotti in 1997 first posited the polyubiquitination of the NKA, identifying a potential regulatory mechanism involving the α1-and α2-subunits within COS-7 cells (Coppi and Guidotti, 1997b). This initial observation set the stage for understanding the complex interplay between ubiquitination, endocytosis, and degradation of the NKA under conditions of cellular stress, particularly hypoxia.

Thevenod and Friedmann later elucidated the role of oxidative stress in the degradation of the NKA, highlighting the protective role of proteasomal and lysosomal inhibitors (Thévenod and Friedmann, 1999). NKA degradation could be prevented by proteasomal and lysosomal inhibitors, as well as with a defective E1 enzyme, suggesting that the NKA is ubiquitinated, but that degradation occurs in the lysosome instead of the proteasome (Comellas et al., 2006). The internalization of ubiquitinated NKA is mediated by the clathrin-AP2 complex to facilitate endocytosis, representing a critical cellular mechanism for the regulation of membrane protein trafficking (Mukhopadhyay and Riezman, 2007; Chen et al., 2006). Studies have reported that the endogenous α1 subunit of the NKA was co-immunoprecipitated with the endogenous E3 ubiquitin ligases—zinc and ring finger domain containing protein 2 (ZNRF2) and its sister protein ZNRF1, indicating that ZNRF1 and ZNRF2 are responsible for ubiquitination of the α1 subunit of the NKA (Hoxhaj et al., 2012).

Kaneko M. et al. Discovered that ring finger protein 183, a RING finger protein family member instrumental in regulating the transcription of proteins crucial for hypertonic adaptation, interacts specifically with the α1 and β1 subunits of the NKA (Okamoto et al., 2020). This interaction leads to the selective ubiquitination of the β1 subunit, which in turn triggers the degradation of both subunits. While, N-*myc* downstream-regulated gene 2 (NDRG2), a member of the NDRG family that have multiple functions in cell proliferation, differentiation, and stress responses, is known to inhibit the ubiquitination of the β1 subunit, thus preventing its degradation (Takarada-Iemata, 2021).

## 6 Glutathionylation

Glutathionylation is the reversible conjugation of the tripeptide glutathione to protein cysteine residues in a mixed disulfide (Hershko and Ciechanover, 1998). This process has been discovered to be a regulatory factor for a diverse array of protein groups (Dalle-Donne et al., 2007). Under certain circumstances, glutathionylation can occur directly without the involvement of enzymes. This non-enzymatic glutathionylation typically happens under conditions of oxidative stress within the cell, where the thiol groups of protein cysteine residues are oxidized and form a disulfide bond with the thiol group of glutathione. This direct chemical interaction is reversible and can respond to changes in the cell’s redox status (Dalle-Donne et al., 2007). However, there is also evidence suggesting that specific enzymes may be involved in regulating the dynamic process of glutathionylation. For instance, glutathionylation is reversed enzymatically by glutaredoxins, thioredoxins and sulfiredoxin, and can be removed non-enzymatically by disulfide exchange with glutathione (Howie et al., 2013; Dalle-Donne et al., 2007; Musaogullari and Chai, 2020).

The glutathionylation of NKA indeed requires the involvement of enzymes (Petrushanko et al., 2012). Recent reports have identified the α subunit can be glutathionylated at cysteine residues 454, 458, 459, and 244 in purified enzyme preparations and in the hypoxic rat heart, linking these modifications to the suppression of the NKA’s catalytic activity (Petrushanko et al., 2012). Deglutathionylation of the α subunit catalyzed by glutaredoxin or dithiothreitol restoration of the NKA activity (Petrushanko et al., 2012; Fuller et al., 2003).

Regarding each subtype, the α1 subunit’s S-glutathionylation was specifically enhanced by exposure to oxidized glutathione, a condition that did not affect the β1 subunit, and interestingly, the α2 subunit exhibited greater sensitivity to oxidized glutathione compared to α1. Additionally, treatment with dithiothreitol, a reducing agent, diminished the baseline level of S-glutathionylation of the α1 subunit (Petrushanko et al., 2012). Moreover, the α2 subunit, which is more sensitive to such modifications, contains Cys236 that may serve as an additional target for S-glutathionylation, along with Cys244, further contributing to the regulatory effects on NKA activity (Petrushanko et al., 2012).

The β subunit of the cardiac Na+ pump is known to be modulated by glutathionylation (Figtree et al., 2009). The functional effect of β1-subunit glutathionylation is to destabilize the interaction between cardiac α and β subunits, and decrease the Vmax of the Na+ pump. Glutathionylation of the β1 subunit at Cys46, a site also subject to palmitoylation (Kang et al., 2008), contributes to E2-E1 conformational change of Na+ pump (Figtree et al., 2012), thereby leading to the inhibition of Na+ pump activity (Figtree et al., 2009). FXYD proteins, particularly FXYD1 and 3, are considered to be capable of reversing the inhibition of NKA caused by the β1 glutathionylation (Liu et al., 2022; Bibert et al., 2011). FXYD proteins possessing the reactive cysteine residue facilitate deglutathionylation of the β1 subunit, thus protecting against oxidative inhibition of the NKA (Bibert et al., 2011).

Ouabain, which stabilizes the pump in its E2 conformation, decreases the vulnerability of β1 subunit to glutathionylation (Liu et al., 2012). The physiological stimuli angiotensin II and forskolin, which emulate the activation of the β1 adrenergic receptor and subsequent stimulation of adenyly cyclase/PKA, have been shown to inhibit the Na+ pump. This inhibition occurs through the mechanism involving NADPH oxidase and is further augmented by elevating the glutathionylation of the β1 subunit (Figtree et al., 2009; White et al., 2010). Conversely, activation of the β3 adrenergic receptor stimulates the NKA through a decrease in glutathionylation of the β1 subunit (Bundgaard et al., 2010). This process is dependent on nitric oxide synthase (Bundgaard et al., 2010), establishing a counterbalancing mechanism to the inhibition previously described. The enhancement of FXYD1 glutathionylation is driven by the same signaling pathways that also facilitate glutathionylation of the β1 subunit, but FXYD1 glutathionylation is suggested to be downstream of β1 subunit glutathionylation (Bibert et al., 2011).

Besides those summarized in Table 1, the NKA can undergo other types of PTMs. Liu, J. et al. Suggested that carbonylation of the NKA α1 subunit may represent a novel regulatory mechanism of NKA signaling (Yan et al., 2016). The direct carbonylation modification of Pro224 in the rat α1 subunit (as shown in Figure 1) determines ouabain-mediated NKA signal transduction and the subsequent regulation of Na+ transport in the renal proximal tubule (Yan et al., 2016). The α1 and α2 subunits of the NKA are also susceptible to S-nitrosylation (Jaffrey et al., 2001). The potential role of this PTM in the pump’s physiological regulation merits further investigation.

## 7 Crosstalk between different PTMs of NKA

However, the various PTMs experienced by the subunits of NKA are not mutually exclusive but often accompanied by crosstalk, making the regulatory network of NKA more refined and complex to meet the needs of physiological processes. As summarized in Table 2, FXYD1 is subject to crosstalk regulation involving both phosphorylation and palmitoylation. Phosphorylation of FXYD1 at Ser68 rapidly promotes its palmitoylation. Phosphorylation of FXYD1 at Ser68 by PKA enhances the mobility of helix 4, which in turn increases the accessibility of the cysteine residues in helix three to the palmitoyl acyltransferase enzymes, promoting FXYD1’s palmitoylation (Tulloch et al., 2011; Fuller et al., 2013). Palmitoylation of FXYD1 was also reported to be promoted by PKC phosphorylation and to be required for inhibition of the NKA activity (Tulloch et al., 2011).

**TABLE 2:** PTMs crosstalk of NKA: subunits, enzymes, sites, and functional impact.

PTMs crosstalk	Subunits	Modification sites	Functions on NKA
Phosphorylation- palmitoylation	FXYD1	Ser68 (Tulloch et al., 2011; Fuller et al., 2013)	Inhibition (Tulloch et al., 2011)
Phosphorylation- ubiquitination	α1	Ser18 (Dada et al., 2007)	Endocytosis of NKA (Dada et al., 2007)
Palmitoylation- glutathionylation	β1	Cys46 (Howie et al., 2013)	Inhibition (Kang et al., 2008; Figtree et al., 2009)

The existence of FXYD1 aids in the removal of glutathione from Cys46 of the β1 subunit, while simultaneously enabling the glutathionylation of FXYD1 at Cys42, a cysteine residue in FXYD1 that could also undergo palmitoylation (Bibert et al., 2011). FXYD1 may be a pump activator (through glutathionylation) or inhibitor (through palmitoylation) depending on the state of Cys42, which is determined by the phosphorylation status of FXYD1, adrenergic state of the tissue and redox state of the cell (Howie et al., 2013).

Reports also show that preventing the phosphorylation of α1 subunit of NKA by PKC-ζ at Ser18 during hypoxia inhibits ubiquitination, indicating that ubiquitination is dependent on phosphorylation (Dada et al., 2007).

## 8 PTMs of NKA in different conformations

NKA has various conformations: E1-state (induced by Na+), E2-state (induced by K+), E2P-state (induced by Mg2+ +Pi) and E2P-OBN (induced by Mg2+ +Pi and ouabain) (Morth et al., 2007; Kaplan, 2002; Petrushanko et al., 2007; Kanai et al., 2013). Each of these conformations plays a vital role in the generation of action potentials in nerve and muscle cells, and the regulation of cell volume and pH. PTMs of the NKA also have a regulatory interplay with its conformational changes.

The glutathionylation of NKA α subunit in different conformations warrants in-depth discussion, as it may provide insights into how conformational changes affect the efficiency of PTMs. The level of glutathionylation is highest in the E1 conformation of NKA α1 subunit, due to the most availability of cysteine residues for glutathionylation in this state, compared to E2, E2P and E2P-OBN conformation (Poluektov et al., 2019). The NKA a1 subunit contains 15 cysteine residues in the cytosolic portion of the protein, among which 12 can be glutathionylated (Petrushanko et al., 2012; Dergousova et al., 2017). The number of cysteines available to the solvent was found to vary in different NKA conformations, with 15, 10 or nine residues accessible in the E1, E2 and E2P-OBN conformations, respectively. During transition from E1P to E2P or from E2P to E2P-OBN states, the Cys204, Cys242, Cys336 and Cys367 become unavailable for glutathionylation. In the E2P-OBN conformation, Cys656 also becomes unavailable (Poluektov et al., 2019). This unavailability accounts for the observed decrease in glutathionylation levels in the E2, E2P and E2P-OBN conformation of NKA.

Conversely, PTMs also induce conformational changes of the NKA. Glutathionylation of the β1 subunit at Cys46, a site also subject to palmitoylation (Kang et al., 2008), contributes to E2-E1 conformational change of Na+ pump (Figtree et al., 2012), thereby leading to the inhibition of Na+ pump activity (Figtree et al., 2009). Phosphorylation of the α subunit leads to conformational changes in the transmembrane segments H5 and H6 hairpin, which in turn cause alterations in the cation-binding site conformation and the consequent cation translocation (Mikhailova et al., 2002).

## 9 Conclusion and discussion

### 9.1 Phosphorylation of NKA: Site-specific regulation and physiological significance

Phosphorylation constitutes the most extensively characterized PTM of NKA, with at least nine experimentally validated sites identified across the α1, α2, and α3 subunits, as well as on FXYD1 and FXYD2 (summarized in Table 1). Among these, the α1 subunit harbors the largest number of confirmed sites (Ser18/20/943, Tyr10/537), followed by α2 (Ser364/461/464, Tyr537) and α3, whose phosphorylation sites remain to be precisely mapped. The regulatory kinases include PKA, PKC (including ζ and ε isoforms), Src-family tyrosine kinases, and the receptor tyrosine kinase Agrin, whereas dephosphorylation is mediated predominantly by PP1 and PP2A, with calcineurin also implicated in specific tissues.

The functional consequences of individual phosphorylation events are remarkably site-specific and context-dependent. Phosphorylation of α1 at Ser18 and Ser20 by PKC-ζ triggers clathrin-dependent endocytosis, leading to pump removal from the plasma membrane, whereas phosphorylation at Ser943 by PKA exerts an inhibitory effect without altering membrane abundance. Conversely, phosphorylation at Tyr10 mediates the stimulatory effect of insulin on NKA activity in renal proximal tubules, linking this modification to enhanced sodium reabsorption, and Tyr537 phosphorylation is required for AP-2 binding and endocytosis. The α2 subunit, predominantly expressed in heart and skeletal muscle, is regulated by PKC-ε-mediated phosphorylation at Ser464 under stress conditions, while Ser364 phosphorylation leads to ER retention. For the regulatory subunit FXYD1, PKA- and PKC-mediated phosphorylation at Ser68, Ser63, and Thr69 relieves its intrinsic inhibition of the pump, increasing both Na^+^ affinity and Vmax, whereas dephosphorylation by PP1 and PP2A restores the inhibitory state.

The physiological relevance of this multilayered phosphorylation network is perhaps best exemplified by the opposing actions of natriuretic and antinatriuretic signals in the kidney. Dopamine, via PKC-dependent phosphorylation of α1 at Ser18, promotes NKA internalization and reduces sodium reabsorption, contributing to natriuresis and blood pressure reduction. Conversely, insulin, acting through Src-mediated Tyr10 phosphorylation, enhances NKA activity and sodium retention, counterbalancing the dopaminergic effect. The α-adrenergic agonist oxymetazoline, through calcineurin-mediated dephosphorylation, stimulates NKA activity at low intracellular sodium, further illustrating the integration of phosphorylation and dephosphorylation in sodium homeostasis. In the heart, FXYD1 phosphorylation by PKA mediates the β-adrenergic-induced activation of NKA, limiting cardiomyocyte Na^+^ overload and thereby protecting against arrhythmias.

Collectively, the diversity of phosphorylation sites, the specificity of their modifying enzymes, and their distinct physiological outcomes establish NKA phosphorylation as a dynamic and integrative regulatory mechanism that fine-tunes ion transport in response to hormonal, metabolic, and hemodynamic cues. Dysregulation of these phosphorylation events has been implicated in hypertension, heart failure, and salt-sensitive kidney disease.

#### 9.1.1 Glycosylation: Roles in trafficking, stability, and adhesion

Unlike phosphorylation or palmitoylation of NKA, glycosylation does not directly regulate ion transport. Rather, its primary functions are indirect, operating at the levels of protein folding, intracellular trafficking, membrane stability, and intercellular adhesion.

Experimental evidence indicates that removal of N-glycans from the β1 or β3 subunit does not significantly alter the catalytic activity of the pump once it reaches the plasma membrane (Beggah et al., 1997; Laughery et al., 2003; Takeda et al., 1988). For the β2 subunit, glycosylation is essential for ER exit and α-subunit association; its absence leads to retention and failed assembly (Tokhtaeva et al., 2010). These findings establish glycosylation primarily as a quality control and trafficking determinant.

In addition, glycosylation of β1 and β2 mediates homophilic trans-interactions required for cell adhesion, a function independent of ion pumping (Vagin et al., 2006; Roldán et al., 2022). For FXYD7, O-glycosylation stabilizes the protein without affecting pump modulation (Béguin et al., 2002). Most conclusions derive from heterologous systems; whether glycosylation is dynamically regulated *in vivo* and whether it contributes to disease remain largely unknown.

#### 9.1.2 Glutathionylation: Redox-sensitive inhibition of NKA

Glutathionylation of NKA has emerged as a redox-sensitive regulatory mechanism that inhibits pump activity under oxidative stress. To date, five cysteine residues have been experimentally identified as glutathionylation sites on the catalytic α subunit: Cys244, Cys454, Cys458, and Cys459 on α1, and Cys236 and Cys244 on α2. Among these, Cys244 is shared by both α1 and α2, suggesting a conserved redox-sensing function across isoforms. The β1 subunit is also susceptible at Cys46, a site additionally subject to palmitoylation.

The functional consequence of glutathionylation is uniformly inhibitory, but the underlying mechanisms differ by subunit. On the α subunit, glutathionylation directly suppresses catalytic activity, with deglutathionylation by glutaredoxin restoring pump function (Figtree et al., 2012). On the β1 subunit, glutathionylation at Cys46 destabilizes α–β interaction and reduces Vmax, an effect that can be reversed by FXYD1 and FXYD3, which facilitate deglutathionylation of β1 (White et al., 2010).

Importantly, the extent of glutathionylation is highly context-dependent. Under basal physiological conditions, the modification level is relatively low. Oxidative stress, such as hypoxia/reoxygenation, exposure to oxidized glutathione, or stimulation with angiotensin II or forskolin, markedly elevates glutathionylation at these sites, correlating with reduced pump activity (Liu et al., 2022; Yan et al., 2016). The α2 subunit exhibits greater sensitivity to oxidized glutathione than α1, suggesting isoform-specific redox susceptibility. Conversely, β3-adrenergic receptor activation counteracts this inhibition by decreasing β1 glutathionylation through a nitric oxide-dependent pathway (Bundgaard et al., 2010). Conformational state also modulates accessibility: glutathionylation is most efficient in the E1 conformation, whereas ouabain-stabilized E2 conformation reduces susceptibility (Bundgaard et al., 2010; Poluektov et al., 2019).

In summary, glutathionylation constitutes a reversible redox switch that downregulates NKA activity under oxidative conditions, with distinct sites on α and β subunits contributing through different mechanisms. This modification may serve a protective role by limiting ATP consumption during transient oxidative stress, whereas persistent glutathionylation under chronic oxidative conditions could contribute to ion dyshomeostasis in ischemic heart and kidney disease.

### 9.2 Future perspectives

Despite significant progress in identifying PTMs on NKA subunits, several fundamental knowledge gaps remain. First, the functional significance of individual PTM sites, particularly those identified by proteomic screens but lacking functional validation, remains largely unexplored. Second, the crosstalk between different PTMs, such as the interplay between palmitoylation and phosphorylation on FXYD1 or between glutathionylation and ubiquitination on the α subunit, is only beginning to be understood and requires systematic investigation. Third, the tissue- and disease-specific PTM patterns of NKA have not been characterized, nor is it known whether these patterns change dynamically in response to physiological stimuli or pathological insults. Fourth, the reversibility and temporal dynamics of most NKA PTMs remain poorly defined, limiting our understanding of their roles as acute regulatory switches versus long-term stability determinants.

Advances in emerging technologies are poised to address these gaps. Quantitative phosphoproteomics and global palmitoylation profiling have already enabled high-confidence identification of novel modification sites, and single-cell proteomic approaches may soon reveal cell-type-specific PTM landscapes within complex tissues. Structural biology, particularly cryo-electron microscopy, offers the opportunity to visualize how site-specific modifications alter NKA conformation at atomic resolution. CRISPR-based editing of PTM sites or their corresponding enzymes enables functional studies in physiologically relevant models, while AI-assisted drug discovery could accelerate the design of small molecules that modulate PTM-enzyme interactions with high selectivity.

Translating these insights into clinical applications faces substantial challenges. Therapeutic strategies targeting PTM enzymes must overcome issues of off-target effects, given the shared catalytic mechanisms among many kinases, phosphatases, and acyltransferases. Achieving tissue-specific delivery of PTM-modulating agents remains a major obstacle, particularly for kidney- or heart-specific NKA regulation. Furthermore, validation in appropriate animal models will be essential before human trials can be considered. These models should include genetically engineered mice with PTM site mutations and disease-relevant models of hypertension, heart failure, and ischemia.

Beyond therapy, PTM profiling holds significant promise for diagnostics and precision medicine. Distinct PTM patterns may serve as biomarkers for disease stratification, enabling identification of patients who are most likely to benefit from specific PTM-targeted interventions. Moreover, longitudinal monitoring of PTM profiles could inform prognosis and guide treatment decisions in NKA-associated diseases.

A comprehensive understanding of the functional significance of individual modifications, their dynamic interplay, and their regulation across tissues and diseases will not only illuminate fundamental mechanisms of ion transport regulation but also lay the groundwork for novel diagnostic and therapeutic strategies.
